# The mediating role of psychological flexibility in the association of autistic-like traits with burnout and depression in medical students during clinical clerkships in Japan: a university-based cross-sectional study

**DOI:** 10.1186/s12888-023-04811-y

**Published:** 2023-05-01

**Authors:** Takafumi Watanabe, Tatsuo Akechi

**Affiliations:** Department of Psychiatry and Coginitive-Behavioral Medicine, Nagoya City University Graduate School of Medical Sciences, Nagoya City, Aichi Japan

**Keywords:** Medical students, Burnout, Depression, Autistic-like traits, Psychological flexibility

## Abstract

**Background:**

Burnout and depression among medical students is linked to serious problems that require appropriate solutions. Subthreshold autism traits or autistic-like traits (ALTs) may be possible factors associated with burnout and depression. The effectiveness of acceptance and commitment therapy (ACT) for burnout and depression has been widely reported. The treatment aims to improve psychological flexibility, a concept indicating engagement in personal value-based behaviors without avoiding uncomfortable private experiences. This study examined whether ALTs were associated with burnout or depression among medical students during clinical clerkships in Japan, and then investigated what psychological flexibility processes might mediate these associations.

**Methods:**

A cross-sectional survey was administered to 284 medical students at Nagoya City University School of Medical Sciences who had been in clinical clerkships for 10 months or longer. Linear multiple regressions were performed with each burnout factor or depression as the outcome variable using validated tools measuring burnout (Maslach Burnout Inventory), anxiety and depression (Hospital Anxiety and Depression Scale), ALT (Autism-Spectrum Quotient Japanese version-21), and psychological flexibility processes (Cognitive Fusion Questionnaire-7 and Valuing Questionnaire). Additionally, a mediation analysis was conducted using structural equation modeling.

**Results:**

A linear multiple regression analysis that controlled for age and gender found that ALTs were significantly associated with lower personal accomplishment, a factor of burnout, and depression. Lower personal accomplishment was also associated with males and lower progress toward values of the psychological flexibility process. Depression was also associated with males and higher cognitive fusion, lower progress towards values, and higher obstruction to values of the psychological flexibility process. Surprisingly, emotional exhaustion and depersonalization were not significantly associated with ALTs. The mediation analysis revealed that the relationship between ALTs and personal accomplishment was partially mediated by a process of progress toward values, while the relationship between ALTs and depression was partially mediated by both processes of progress toward values and cognitive fusion.

**Conclusions:**

ALTs were significantly associated with lower personal accomplishment of burnout and depression among medical students in clinical clerkships. Consideration should be given to the psychological flexibility processes that focus on interventions targeting psychological flexibility for medical students with ALTs to reduce burnout and depression.

**Supplementary Information:**

The online version contains supplementary material available at 10.1186/s12888-023-04811-y.

## Introduction

Burnout and depression among medical students have serious consequences, including suicidal ideation [1, 2], dropping out [3], substance abuse, poor sleep quality, decreased work engagement [4], decreased professionalism [5], decreased academic effectiveness, poor mental health overall [6, 7]. Unfortunately, such students also tend not to seek help for their mental health problems [8]. Burnout worsens around the clinical year of medical school, with multiple reports that indicate higher rates of burnout in the clinical year than in preclinical years [7, 8]. Burnout and depression are also associated with poor quality of patient care, absenteeism, and high turnover, which has a significantly negative impact on hospital productivity. It is, therefore, essential to address these issues [9–13].

Burnout is a psychological syndrome that appears as a response to chronic interpersonal stressors at work and is characterized by three factors: (1) emotional exhaustion (the inability to respond in an emotionally appropriate way due to fatigue); (2) depersonalization (responding to subjects of work coldly or in a detached way); (3) a decreased sense of personal accomplishment (a reduced sense of accomplishment as a professional) [14].

The International Classification of Diseases, 11th edition (ICD-11) defines burnout as a syndrome resulting from chronic workplace stress that is not well managed [14]. Since burnout is work-related and context-dependent, whereas depression is more general and context-independent, research has demonstrated that the two constructs, while interrelated, are indeed distinct [15].

Numerous studies have reported on factors associated with burnout and depression in medical students [2, 16–21]. Demographic factors also play a part such as gender, age, race, personal stress experiences (e.g. family or economic problems), a stressful learning environment, or being enrolled in a rigorous program (e.g. a clinical year) [17, 18, 21]. Personality factors such as one’s Big Five Personality score and psychological flexibility factors, a concept that indicates the avoidance of unpleasant internal experiences and disengagement from personal value-based behaviors, also make an impact as well [22–25].

Autism Spectrum Disorder (hereafter, autism), characterized by impairments in social interaction, communication, and behavioral flexibility, is found in 0.76% of the global population [26]. Autistic people experience mental health problems at higher rates than non-autistic people. A recent meta-analysis found a lifetime prevalence of 11% for depressive disorders among adults with autism [27]. In addition to autistic people, many others exhibit subthreshold autism traits or autistic-like traits (ALTs), i.e., problems in characteristics of social communication behaviors, perceptions of others and the self, and adaptations to the environment that do not meet the formal criteria for autism [28]. ALT has been reported to be associated with burnout and depression among hospital pharmacists [29, 30]. A previous study in Japan also reported that ALTs were significantly associated with clinically depression among medical students in clinical clerkships [31]. However, the association between ALT and burnout among Japanese medical students has not been reported.

Acceptance and commitment therapy (ACT), a psychotherapy that targets the facilitation of psychological flexibility, has been used and reported to be effective in reducing burnout and depression among a variety of healthcare professionals as well as medical students [23, 32–35]. Psychological flexibility is a concept that indicates (1) engagement in behavior that is motivated by an individual's values and (2) not avoiding unpleasant private (or internal) experiences. Value-based behavior is purposeful action, moving in a direction that one considers important to oneself, even in the presence of unpleasant private experiences (e.g., thoughts, feelings, memories, and impulses). Experiential avoidance is the effort to change, repress, or eliminate unpleasant private experiences [36]. Experiential avoidance usually provides short-term benefits, but in the long term, it can misdirect important life directions for the self [37]. For example, a highly experiential-avoidant medical student might put off important work because engaging with work causes discomfort. A medical student who is disconnected from their values may work mechanically without engaging in otherwise important values such as helping others, including patients, being loving toward others, and other prosocial behaviors.

Medical students experienced high infection risk, significant lifestyle changes, strict confinement, and disrupted education during the COVID-19 pandemic. Likely as a result, they were extremely vulnerable to mental health problems such as burnout, anxiety, depression, and insomnia [20, 38–40]. In Japan, anxiety and depression have increased among medical students after the pandemic-induced nationwide emergency [39]. Improving the mental health of medical students after COVID-19 is a critical issue.

One important aspect of addressing this issue is enhancing psychological flexibility, but we know little about the characteristics of psychological flexibility in medical students with ALTs suffering from burnout and depression. This study aimed to examine whether ALT is associated with burnout or depression among medical students in clinical clerkships in Japan. We also aimed to identify core processes of psychological flexibility that mediate the relationship between ALTs and burnout or depression. Lastly, we evaluated pre- and post-COVID-19 differences in student mental health problems, including burnout, anxiety, and depression.

## Methods

### Design and setting

This was a descriptive cross-sectional study conducted at Nagoya City University School of Medical Sciences, one of Japan’s small urban universities.

### Participant recruitment

At the School of Medical Sciences, students begin their clinical clerkships in January of their fourth year at the university-affiliated hospital (they become fifth-year students in April of the same year), and finish in October of their sixth year. All 284 medical students (147 men and 79 women) who had been in clinical clerkships for at least 10 months from November 2019 to October 2022 and were still in clinical practice at the time of the study were invited to participate in an online survey. There was no prior information on the participants’ neurodiversity or mental health conditions such as autism, attention-deficit/hyperactivity disorder, anxiety disorder, or clinical depression. Since April 2020, the COVID-19 pandemic has substantially restricted face-to-face practice of the clinical clerkship curriculum on hospitalized patients. The study was conducted following the guidelines described in the “Strengthening the Reporting of Observational Studies in Epidemiology” statement. Procedures were approved by the Institutional Review Board of Nagoya City University Graduate School of Medical Sciences. All participants provided consent before completing the survey.

### Measures

#### Sociodemographic characteristics

Demographic and occupational information included participants gender, age, history of enrollment in other undergraduate courses, marital and parental status, cohabitants, extracurricular activity, part-time employment, and alcohol use (i.e., consuming alcohol more than three times per week).

#### Outcome variables

##### Burnout

The Maslach Burnout Inventory (MBI), a 22-item self-administered rating instrument is considered the gold standard for measuring burnout. This scale was administered in the current study [41]. The MBI was purchased for licensed use from Mindgarden Inc. Respondents indicate the frequency of each item on a 7-point Likert scale ranging from 0 (*not at all*) to 6 (*every day*). The MBI consists of three factors: (1) emotional exhaustion (MBI-EE), (2) depersonalization (MBI-DP), and (3) personal accomplishment (MBI-PA). The total score for each of the three factors was recorded. A high score on the MBI-EE subscale indicates an inability to cope with occupational emotional stressors. A high score on the MBI-DP subscale indicates negative feelings and loss of interest in patients’ health. A low score on the MBI-PA subscale indicates a decreased sense of accomplishment and affirmation in the healthcare provider-patient interaction. The MBI has been used extensively in studies involving physicians [42–44] and medical students [1, 45, 46]. The good reliability and validity of the Japanese version were demonstrated in previous research [47]. Criteria for burnout were based on large U.S. studies. High emotional exhaustion was defined as an MBI-EE score of ≥ 27, high depersonalization as an MBI-DP score of ≥ 10, low personal accomplishment as an MBI-PA score of ≤ 33, and overall burnout as an MBI-EE score of ≥ 27 and/or an MBI-DP score of ≥ 10 [1, 3, 48]. In the present study, the Cronbach's alpha reliability coefficients for the three MBI subscales were satisfactory (MBI-EE α = 0.90, MBI-DP α = 0.78, MBI-PA α = 0.81).

##### Anxiety and depression

In this study, the Hospital Anxiety and Depression Scale (HADS), developed for clinical populations, was employed to measure anxiety and depression [49]. This scale has been widely used in previous studies among students in general and medical students [2, 24, 50]. The HADS comprises two subscales—on anxiety and depression—including seven items each with a 4-point Likert scale. Each subscale score ranged from 0–21, and the total score ranged from 0–42. A higher total score indicated greater anxiety and depression. The psychometric properties of the Japanese version of the questionnaire have been validated. Cutoffs for the HADS Anxiety subscale (HADS-A), HADS Depression subscale (HADS-D), and HADS total (HADS-Total) scores for screening major depressive disorder were 7/8 (sensitivity 94.1%, specificity 87.7%), 10/11 (sensitivity 82.4%, specificity 95.1%) and 19/20 (sensitivity 82.4%, specificity 96.3%), respectively [51]. Medical students were categorized as highly anxious with an HADS-A score of ≥ 8, highly depressed with an HADS-D score of ≥ 11, and highly anxious-depressed with an HADS-Total score of ≥ 20 [51]. Cronbach's alpha reliability coefficients were satisfactory for the three scales (HADS-A α = 0.76, HADS-D α = 0.70, HADS-Total α = 0.81).

#### Explanatory variables

##### Autistic-like traits (ALTs)

To measure autistic-like traits, the Japanese version of the Autism-Spectrum Quotient (AQ-J)-21, was used. The AQ-J-21 is a shortened version of the Japanese version of the AQ, which was originally a 50-item self-administered scale. It is used worldwide to measure the degree to which adults with intelligence scores in the normal range have autism spectrum traits [52]. It has higher sensitivity and specificity than the 50-item AQ-J [53]. It discriminates autistic people from a control group selected from the general population using a cutoff value of 12 points or more [53]. In this study, this criterion was used to determine the presence of autistic traits. Cronbach’s alpha reliability coefficient was satisfactory for the AQ-J-21 (α = 0.70).

##### Psychological flexibility

Psychological flexibility was measured using the Valuing Questionnaire (VQ), and the Cognitive Fusion Questionnaire-7 (CFQ-7). The VQ is a 10-item measure of the values process, with two subscales: progress in valued living (VQ-P) and obstruction to valued living (VQ-O) [54]. Each item is rated on a 7-point Likert scale. Higher scores on the progress subscale indicate higher living, while higher scores on the obstruction subscale indicate greater disturbance in valued living. The CFQ-7 is a 7-item measure of cognitive fusion that assesses the fusion of thoughts and meaning [55]. Each item is rated on a 7-point Likert scale. Higher scores indicate higher cognitive fusion. Both the VQ and the CFQ-7 were shown to have reliability and validity in the Japanese version [56, 57]. The VQ was selected as a simplified assessment of value-based behavior and experiential avoidance, whereas the CFQ-7 was selected because medical students were expected to be more verbally competent and more likely to be caught up in negative thoughts. Cognitive fusion is regarded as the indicator of experiential avoidance through thinking [36]. Cronbach’s alpha reliability coefficient was acceptable for the two scales (CFQ-7 α = 0.91, VQ-P α = 0.83, VQ-O α = 0.57).

### Data collection

Data was collected anonymously through an online survey using Google Forms. All survey questions were presented together when medical students gathered for several lectures that took place more than 10 months after the clinical clerkship began. Gift certificates worth 500 yen were offered as an incentive for responding to the survey.

### Statistical analysis

Bivariate analysis was conducted to compare changes in each variable before and after the COVID pandemic. Categorical variables were evaluated for significant differences using the chi-square test. Continuous variables were evaluated using t-tests or Mann–Whitney U tests. Subsequently, bivariate correlations were examined. Multiple linear regression analyses were then conducted to identify factors explaining the greatest variation in burnout (emotional exhaustion, depersonalization, and personal accomplishment) and depression. After controlling for gender and age as potential confounding factors, independent associations between the outcome variables of burnout as well as anxiety/depression and the explanatory variables (i.e., ALTs, psychological flexibility) were examined [58]. These statistical analyses were performed using EZR, a graphical user interface for R version 3.6.1. Structural equation modeling was utilized to investigate the mediating effects of psychological flexibility on the relationship between ALTs and burnout and depression using Muthén & Muthén Mplus 8.8. The magnitude of the indirect effect of ALTs on burnout and depression with psychological flexibility as the mediator was estimated using the bias-corrected bootstrap method with 1000 replications to obtain bootstrap 95% confidence intervals (CIs) [59]. The indirect effect was considered significant if CIs did not include zero. The significance level was set at *p* < 0.05.

## Results

### Sociodemographic characteristics

Of the 284 medical students invited to participate in the study, 79.6% (*n* = 226) responded positively (Table 1). The sample included 147 men (65.0%) and 79 women (35.0%) with a median age of 24.0 years (interquartile range [IQR], 23.0–24.0). The prevalence of high emotional exhaustion, high depersonalization, low personal accomplishment, and overall burnout was 28.8% (*n* = 65, 95% confidence interval [CI], 23.0–35.1), 14.6% (*n* = 33, 95% CI, 10.3–19.9), 87.6% (*n* = 198, 95% CI], 82.6–91.6) and 34.1% (*n* = 77, 95% CI, 27.9–40.6), respectively. The prevalence of high anxiety, high depression, and high anxiety-depression was 50.9% (*n* = 115, 95% CI, 44.2–57.6), 17.7% (*n* = 40, 95% CI, 13.0–23.3), and 23.5% (*n* = 53, 95% CI, 18.1–29.5), respectively. The proportion of ALTs was 27.0% (*n* = 61, 95% CI, 21.3–33.3). A bivariate analysis evaluating differences in subgroup characteristics before and after the COVID-19 pandemic showed a significant decrease in the rate of high depersonalization, overall burnout, high anxiety, depression and anxiety-depression after COVID-19. Additionally, median MBI-EE, DP, PA, HADS-A, HADS-D, and HADS-Total scores significantly decreased (Table 1). There were no statistically significant differences in demographic characteristics or median scores of AQ-21, CFQ-7, VQ-P, or VQ-O. Demographic characteristics and descriptive data categorized by gender are presented as supplementary material (Additional file 1: Table S1). Any gender differences in ALT percentages or median AQ-J-21 scores were not significant.

**Table 1:** Demographic characteristics and descriptive data before and after the COIVID-19 pandemic

Variable	Total number(*n* = 226)	Before COVID (*n* = 61)	After COVID (*n* = 165)	*p*-value^*^
	Number (%)			
Gender (men)	147 (65.0)	38 (62.3)	109 (66.1)	0.711
Gender (women)	79 (35.0)	23 (37.7)	56 (33.9)	
High emotional exhaution^**^	65 (28.8)	23 (37.7)	42 (25.5)	0.101
High depersonalition^**^	33 (14.6)	**17 (27.9)**	**16 ( 9.7)**	**0.001**
Low personal accomplishment^**^	198 (87.6)	52 (85.2)	146 (88.5)	0.668
Overall burnout^**^	77 (34.1)	**29 (47.5)**	**48 (29.1)**	**0.015**
Highly anxious^***^	115 (50.9)	**39 (63.9)**	**76 (46.1)**	**0.025**
Highly depressed^***^	40 (17.7)	**19 (31.1)**	**21 (12.7)**	**0.002**
Highly anxious-depressed^***^	53 (23.5)	**25 (41.0)**	**28 (17.0)**	** < 0.001**
ALT ^a^	61 (27.0)	18 (29.5)	43 (26.1)	0.727
Alcohol consumption more than 3 times/week	29 (12.8)	9 (14.8)	20 (12.1)	0.763
Participation in an extracurricular activity	156 (69.0)	37 (60.7)	119 (72.1)	0.136
Presence of housemates	90 (39.8)	24 (39.3)	66 (40.0)	1
Presence of a part-time job	153 (67.7)	40 (65.6)	113 (68.5)	0.799
History of enrollment in other faculties	15 (6.6)	5 (8.2)	10 (6.1)	0.786
Spouse	4 (1.8)	1 (1.6)	3 (1.8)	1
Parenting	4 (1.8)	1 (1.6)	3 (1.8)	1
	Median [IQR^**^]			
Age	24.00 [23.00, 24.00]	23.00 [23.00, 24.00]	24.00 [23.00, 24.00]	0.335
MBI-EE^b^	17.00 [9.00, 29.00]	**23.00** **[14.00, 31.00]**	**16.00** **[9.00, 28.00]**	**0.037**
MBI-DP^c^	2.00 [0.00, 6.00]	**5.00** **[1.00, 12.00]**	**1.00** **[0.00, 4.00]**	** < 0.001**
MBI-PA^d^	22.00 [15.00, 29.00]	**25.00** **[15.00, 30.00]**	**21.00** **[14.00, 27.00]**	**0.032**
HADS-A^e^	8.00 [5.00, 10.00]	**9.00** **[6.00, 12.00]**	**7.00** **[4.00, 9.00]**	**0.002**
HADS-D^f^	6.00 [4.00, 9.75]	**8.00** **[5.00, 11.00]**	**6.00** **[4.00, 9.00]**	**0.001**
HADS-Total^g^	14.00 [9.00, 19.00]	**19.00** **[12.00, 21.00]**	**13.00** **[9.00, 17.00]**	** < 0.001**
AQ-J-21 h	9.00 [6.00, 12.00]	10.00 [7.00, 12.00]	9.00 [6.00, 12.00]	0.286
CFQ-7 ^i^	24.00 [17.00, 29.00]	26.00 [20.00, 29.00]	24.00 [17.00, 30.00]	0.517
VQ -P^j^	19.00 [15.00, 23.00]	18.00 [15.00, 21.00]	19.00 [15.00, 23.00]	0.342
VQ-O^k^	17.00 [14.00, 21.00]	17.00 [15.00, 20.00]	18.00 [14.00, 21.00]	0.613

^*^ Chi-square tests for categorical data. Mann–Whitney U test for numerical data. Bold text indicates significant difference before and after the COVID-19 pandemic at *p* < 0.05

^**^ MBI-EE score of ≥ 27 was defined as high emotional exhaustion, MBI-DP score of ≥ 10 as high depersonalization, MBI-PA score of ≤ 33 as low degree of personal accomplishment, and MBI-EE score of ≥ 27 and/or MBI-EE score of ≥ 10 as overall burnout

^*^^**^ A score of ≥ 8 on the HADS Anxiety subscale was categorized as highly anxious, a score of ≥ 11 on the HADS Depression subscale as highly depressed, and a total score of ≥ 20 as highly anxious-depressed

^****^ IQR: Interquartile Range

^a^*ALT* Autistic-like trait

^b^*MBI-EE* Maslach Burnout Inventory-Emotional Exhaustion

^c^*MBI-DP* Maslach Burnout Inventory-Depersonalization

^d^*MBI-PA* Maslach Burnout Inventory-Personal Accomplishment

^e^*HADS-A* Hospital Anxiety and Depression Scale-Anxiety subscale score

^f^*HADS-D* Hospital Anxiety and Depression Scale-Depression subscale score

^g^*HADS-Total* Hospital Anxiety and Depression Scale-Total score

^h^*AQ-J-21* Autism-Spectrum Quotient, Japanese version -21

^i^*CFQ-7* Cognitive Fusion Questionnaire-7

^j^*VQ-P* Valuing Questionnaire-Progress

^k^*VQ-O* Valuing Questionnaire-Obstruction

### Correlation between scales

Binary correlations showed that MBI-EE correlated positively with MBI-DP, HADS-A, HADS-D, HADS-Total, CFQ-7, and VQ-O but negatively with VQ-P. MBI-DP was positively correlated with MBI-EE, HADS-A, HADS-D, HADS-Total, and CFQ-7. MBI-PA correlated positively with VQ-P but negatively with HADS-A, HADS-D, HADS-Total, AQ-J-21, and VQ-O.

In terms of the HADS scales, HADS-A correlated positively with HADS-D, HADS-Total, AQ-J-21, CFQ-7, and VQ-O but negatively with VQ-P. HADS-D correlated positively with HADS-Total, AQ-J-21, CFQ-7, and VQ-O but negatively with VQ-P. HADS-Total correlated positively with AQ-J-21, CFQ-7, and VQ-O but negatively with VQ-P.

AQ-J-21 correlated positively with CFQ-7 but negatively with VQ-P. CFQ-7 correlated positively with VQ-O but negatively with VQ-P, while VQ-P was negatively correlated with VQ-O (Table 2).

**Table 2:** Results of correlation analysis

	1	2	3	4	5	6	7	8	9	10
1. MBI-EE^a^	1									
2. MBI-DP ^b^	0.53^**^	1								
3. MBI-PA ^c^	-0.06	0.03	1							
4. HADS-A ^d^	0.36^**^	0.27^**^	-0.16^*^	1						
5. HADS-D ^e^	0.42^**^	0.35^**^	-0.31^**^	0.51^**^	1					
6. HADS-Total^f^	0.45^**^	0.36^**^	-0.27^**^	0.88^**^	0.86^**^	1				
7. AQ-J-21^g^	0.17	0.10	-0.26^**^	0.19^**^	0.24^**^	0.25^**^	1			
8. CFQ-7^h^	0.37^**^	0.20^**^	-0.07	0.54^**^	0.42^**^	0.55^**^	0.14*	1		
9. VQ-P ^i^	-0.23^**^	-0.12	0.39^**^	-0.34^**^	-0.42^**^	-0.44^**^	-0.21**	-0.28**	1	
10. VQ-O ^j^	0.33^**^	0.08	-0.13^*^	0.40^**^	0.35^**^	0.43^**^	0.11	0.50**	-0.27**	1

^*^ *p* < 0.05

^**^ *p* < 0.01

^a^*MBI-EE* Maslach Burnout Inventory-Emotional Exhaustion

^b^*MBI-DP* Maslach Burnout Inventory-Depersonalization

^c^*MBI-PA* Maslach Burnout Inventory-Personal Accomplishment

^d^*HADS-A* Hospital Anxiety and Depression Scale-Anxiety subscale score

^e^*HADS-D* Hospital Anxiety and Depression Scale-Depression subscale score

^f^*HADS-Total* Hospital Anxiety and Depression Scale-Total score

^g^*AQ-J-21* Autism-Spectrum Quotient, Japanese version -21

^h^*CFQ-7* Cognitive Fusion Questionnaire-7

^i^*VQ-P* Valuing Questionnaire-Progress

^j^*VQ-O* Valuing Questionnaire-Obstruction

### Multiple linear regression

A linear multiple regression analysis that controlled for gender and age showed that an MBI-EE score was associated with higher CFQ-7 and VQ-O scores among psychological flexibility, and an MBI-DP score was associated with a higher CFQ-7 score among males. However, neither the MBI-EE score nor the MBI-DP score was significantly associated with ALTs (Table 3). The MBI-PA score was associated with lower ALTs, male gender, and a higher VQ-P score. The HADS-A score was significantly associated with higher CFQ, higher VQ-O scores, and lower VQ-P scores, but not with ALT. Both HADS-D and HADS-Total were associated with higher ALTs, male gender, higher CFQ-7, lower VQ-P, and higher VQ-O. The diagnostic tests showed that the variance inflation factor (VIF) ranged from 1.02 to 1.41 in all regression analyses, indicating that there was no substantial multicollinearity among the explanatory variables.

**Table 3:** Linear multiple regression with burnout, anxiety, and depression as outcome variables

		95% CI^*^				
Variables	β	Lower	Upper	t	*p*-value	Multiple R^2^
MBI-EE^a^						
Constant	-0.382	-19.267	18.504	-0.040	0.968	
Age	0.184	-0.461	0.828	0.562	0.575	
Gender	1.632	-1.687	4.951	0.969	0.333	
AQ-J-21^b^	0.313	-0.109	0.736	1.461	0.145	
**CFQ-7**^**c**^	**0.341**	**0.146**	**0.537**	**3.448**	**0.001**	
VQ-P^d^	-0.228	-0.503	0.048	-1.626	0.105	
VQ-O^e^	**0.469**	**0.084**	**0.854**	**2.399**	**0.017**	0.191
MBI-DP^f^						
Constant	-0.424	-8.940	8.092	-0.098	0.922	
Age	0.077	-0.214	0.367	0.520	0.604	
Gender	**1.560**	**0.063**	**3.056**	**2.054**	**0.041**	
AQ-J-21^b^	0.086	-0.105	0.276	0.886	0.377	
CFQ-7^c^	**0.111**	**0.023**	**0.199**	**2.493**	**0.013**	
VQ-P^d^	-0.065	-0.190	0.059	-1.037	0.301	
VQ-O^e^	-0.029	-0.203	0.145	-0.328	0.743	0.071
MBI-PA^g^						
Constant	4.540	-8.997	18.077	0.661	0.509	
Age	0.390	-0.072	0.852	1.665	0.097	
Gender	**2.667**	**0.289**	**5.046**	**2.210**	**0.028**	
AQ-J-21^b^	**-0.482**	**-0.785**	**-0.179**	**-3.138**	**0.002**	
CFQ-7^c^	0.075	-0.065	0.215	1.052	0.294	
VQ-P^d^	**0.538**	**0.340**	**0.735**	**5.360**	** < 0.001**	
VQ-O^e^	-0.062	-0.338	0.214	-0.441	0.660	0.218
HADS-A^h^						
Constant	0.930	-3.928	5.789	0.377	0.706	
Age	0.089	-0.077	0.255	1.058	0.291	
Gender	-0.051	-0.905	0.803	-0.117	0.907	
AQ-J-21^b^	0.080	-0.029	0.188	1.443	0.151	
CFQ-7^c^	**0.160**	**0.110**	**0.210**	**6.281**	** < 0.001**	
VQ-P^d^	**-0.109**	**-0.180**	**-0.039**	**-3.041**	**0.003**	
VQ-O^e^	**0.117**	**0.018**	**0.216**	**2.321**	**0.021**	0.352
HADS-D^i^						
Constant	1.097	-3.430	5.624	0.478	0.633	
Age	0.117	-0.038	0.271	1.492	0.137	
Gender	**1.485**	**0.690**	**2.281**	**3.680**	** < 0.001**	
AQ-J-21^b^	**0.112**	**0.010**	**0.213**	**2.172**	**0.031**	
CFQ-7^c^	**0.091**	**0.044**	**0.137**	**3.819**	** < 0.001**	
VQ-P^d^	**-0.177**	**-0.243**	**-0.111**	**-5.266**	** < 0.001**	
VQ-O^e^	**0.117**	**0.024**	**0.209**	**2.494**	**0.013**	0.357
HADS-Total^j^						
Constant	2.027	-5.546	9.601	0.528	0.598	
Age	0.206	-0.053	0.464	1.570	0.118	
Gender	**1.434**	**0.104**	**2.765**	**2.124**	**0.035**	
AQ-J-21^b^	**0.191**	**0.022**	**0.361**	**2.224**	**0.027**	
CFQ-7^c^	**0.251**	**0.172**	**0.329**	**6.312**	** < 0.001**	
VQ-P^d^	**-0.286**	**-0.397**	**-0.175**	**-5.098**	** < 0.001**	
VQ-O^e^	**0.233**	**0.079**	**0.388**	**2.980**	**0.003**	0.444

Bold texts indicate statistical significance at *p* < 0.05

^*^CI Confidence interval

^a^*MBI-EE* Maslach Burnout Inventory-Emotional Exhaustion

^b^*AQ-J-21* Autism-Spectrum Quotient, Japanese version-21

^c^*CFQ-7* Cognitive Fusion Questionnaire-7

^d^*VQ* Valuing Questionnaire-Progress

^e^*VQ* Valuing Questionnaire-Obstruction

^f^*MBI-DP* Maslach Burnout Inventory-Depersonalization

^g^*MBI-PA* Maslach Burnout Inventory-Personal Accomplishment

^h^*HADS-A* Hospital Anxiety and Depression Scale-Anxiety subscale score

^i^*HADS-D* Hospital Anxiety and Depression Scale-Depression subscale score

^i^*HADS-Total* Hospital Anxiety and Depression Scale-Total score

### Mediation analysis

**Fig. 1:**
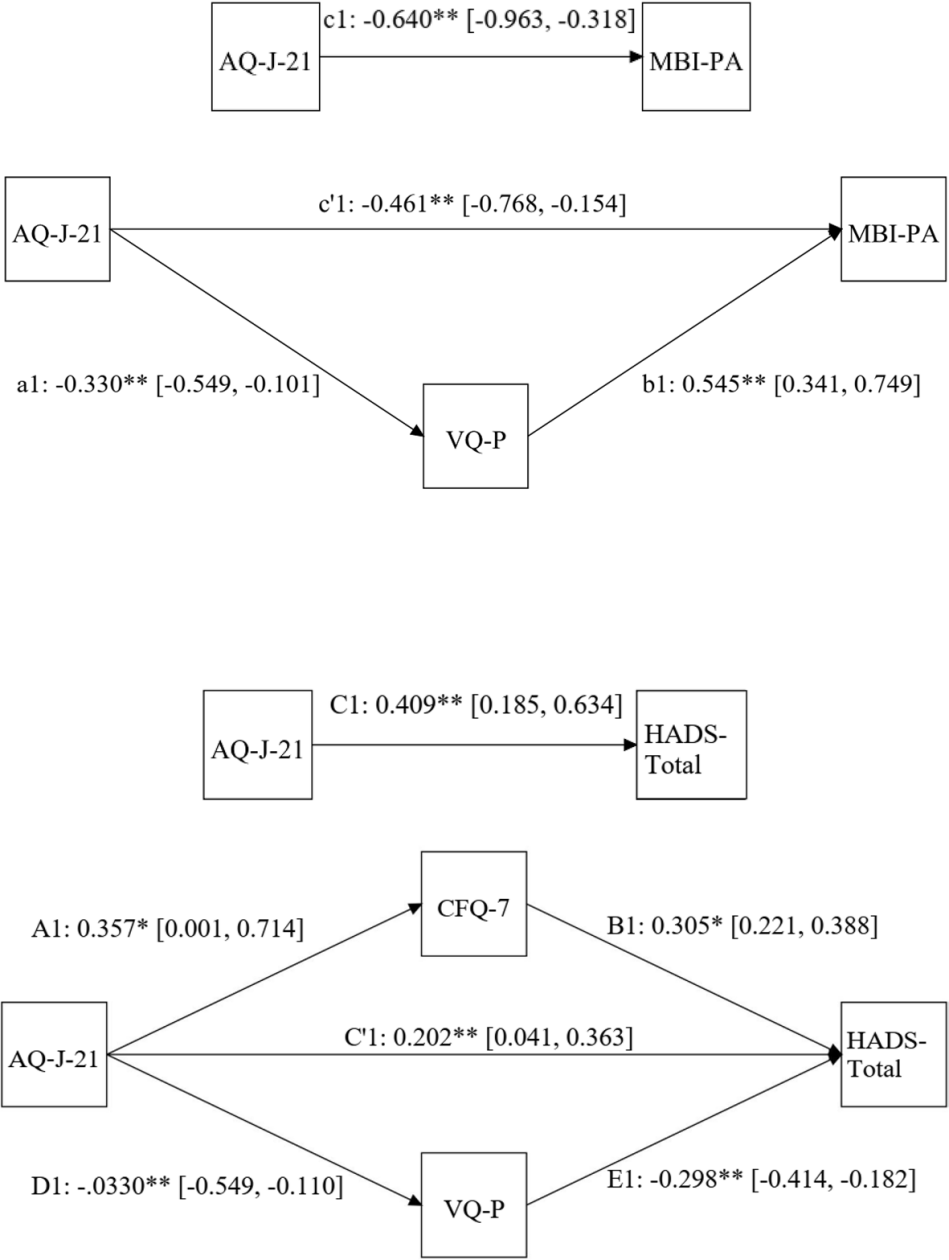
Illustration of the mediation model. Regression coefficients for each path are shown. Boot strap 95% confidence intervals are indicated in square brackets. Path c1 indicates the effect of AQ-J-21 on MBI-PA without an indirect path. Path a1 shows the effect of AQ on VQ-P. Path b1 shows the effect of VQ-P on MBI-PA, with the effect of AQ-J-21 partially excluded. Path c'1 (= c1 - a1 * b1) is the direct effect of AQ-J-21 on MBI-PA. Path C1 indicates the effect of AQ on HADS-Total without an indirect path. Path A1 depicts the effect of AQ on CFQ-7. Path B1 shows the effect of CFQ-7 on HADS-Total, with the effect of AQ partially excluded. Path D1 denotes the effect of AQ on VQ-P. Path E1 indicates the effect of VQ-P on HADS-Total, and the effect of AQ is partially excluded. Pathway C'1 (= C1 - A1 * B1 - D1 * E1) is the direct effect of AQ on HADS-Total. *p < 0.05, **p < 0.01

A mediation analysis using structural equation modeling was conducted to examine what components of psychological flexibility mediate the MBI-PA, HADS-D, and HADS-Total scores, which showed significant associations with ALTs, as outcome variables. ALTs and MBI-PA scores were partially mediated by VQ-P scores (Fig. 1). In the model without mediation, ALTs showed a significant inversely correlated path coefficient (= c1, *p* < 0.05) to the MBI-PA score. In the model with mediation, the bootstrap 95% CI for a1*b1 did not include 0, and all indirect effects in the model were significant. There was also a direct effect, with ALTs showing a significant effect on the MBI-PA score (= c'1, *p* < 0.05). On the other hand, ALTs and HADS-Total scores were partially mediated by CFQ-7 and VQ-P scores. In the model without mediation, ALTs showed a significantly positively correlated path coefficient (= C1, *p* < 0.05) to the HADS score. In the model with mediation, the bootstrap 95% CIs for A1*B1 and D1*E1 did not include 0, and all indirect effects in the model were significant. There was also a direct effect, with ALTs showing a significant effect on the HADS score (= C'1, *p* < 0.05). The HADS-D score also exhibited a similar mediating effect as the HADS-Total score (Additional file 2: Fig. S1).

## Discussion

This study examined the associations between ALTs and each factor of burnout or depression among medical students participating in clinical clerkships. Our results showed that ALT was significantly associated with lower personal accomplishment (a burnout factor), and higher depression. Medical students with ALTs may feel less personally accomplished and more likely to be depressed than medical students without ALTs. This association between ALTs and depression was consistent with our previous report [31]. Surprisingly, ALTs were not associated with emotional exhaustion and depersonalization, the other two burnout factors. This result differs from a previous study that reported an association between ALTs and burnout among hospital pharmacists [30]. One possible reason for this discrepancy is that previous studies used a different scale (subscale of The Professional Quality of Life Scale V) for measuring burnout. We did not use this scale because reliability and validity have not been assessed for the Japanese version. Instead, we chose the MBI, considered to be the gold standard for measuring burnout, and to the best of our knowledge, this is the first study to examine the association between ALTs and each factor of burnout as measured by the MBI. Notably, medical students with ALTs were not more likely to experience emotional exhaustion or depersonalization than those without ALTs, but they were more likely to experience low personal accomplishment. People with ALTs have difficulty identifying, verbalizing, and analyzing emotions [60], suggesting that afflicted medical students may struggle with reporting emotional exhaustion. Diminished personal accomplishment without emotional exhaustion and depersonalization does not strictly fit the definition of burnout and could have been missed.

The mediation analysis revealed that the association between ALTs and personal accomplishment was partially mediated by progress toward values, one of the factors underlying psychological flexibility. This factor, along with cognitive fusion, also partially mediated the association between ALTs and depression. If the motivation of medical students with ALTs is less aligned with the value of "what is important to me" when choosing to become a physician, they may feel less personal accomplishment than medical students without ALTs. In addition to lower engagement in value-based behaviors, ALT medical students with high cognitive fusion are also at a higher risk for depression.

Another factor of psychological flexibility, obstruction of values, did not exert a mediating effect. This factor involves avoidance of unpleasant internal experiences, including feelings and impulses in addition to thoughts [54, 55]. Although medical students with ALTs can logically identify, verbalize, and analyze their thoughts, they have more trouble with emotional experiences. This characteristic suggests that cognitive fusion may be more pronounced than obstruction of values [60]. Interventions for medical students with ALTs, such as ACT to increase psychological flexibility, may therefore be more effective with a stronger focus on specific psychological flexibility processes, e.g., the individual's exploration of his or her own values and cognitive defusion (i.e., releasing the fusion of thought and meaning). We also noted that emotional exhaustion, a burnout factor uncorrelated with ALTs, was positively associated with cognitive fusion and obstruction of values. Similarly, depersonalization was not correlated with ALT either, but was positively associated with cognitive fusion. These results are similar to previous studies and support the idea that increasing psychological flexibility is important to combat burnout for medical students, with and without ALTs [23, 61, 62].

The mean prevalence of emotional exhaustion, depersonalization, and overall burnout was lower than data from a prior review, while mean prevalence of decreased personal accomplishment was higher [19]. Mean prevalence of highly depressed states was also lower in this study than in previous research [2]. However, mean prevalence of overall burnout and depression before the COVID-19 pandemic was consistent with previous reports. Moreover, post-pandemic prevalence of these two conditions was significantly lower than pre-pandemic prevalence. Two systematic reviews reported the post-pandemic prevalence [20, 38]. The first found that mean depression prevalence rose among medical students after the COVID-19 pandemic, while mean burnout prevalence did not change [20]. The second reported that in Japan, the post-pandemic prevalence of depression/anxiety among medical students was significantly lower than in other countries [38, 39]. In our study, over 85% of the medical students had low personal accomplishment before the pandemic. During the post-pandemic period, mean MBI-EE and MBI-DP, and MBI-PA score all decreased significantly.

Clinical clerkships in Japan have long experienced barriers to the introduction of clinical practice, including concerns regarding the safety of medical procedures performed by students, burden to supervising physicians, and the nation’s social medical system [63]. Infrequent clinical practice has caused some medical students to report a low sense of personal accomplishment. For these students, the even lower frequency of clinical practice after the pandemic could have lessened emotional exhaustion, depersonalization, and depression, but also further decreased their sense of personal accomplishment. As noted earlier, increased engagement in value-based behaviors can be effective in addressing low personal accomplishment.The present study found that 27.0% of the surveyed medical students had ALTs, and this percentage did not differ significantly between subgroups before and after the COVID-19 pandemic. In a previous study, 1.9% of college students and 5.5% of hospital pharmacists were reported to have ALTs as measured by AQ [30, 64]. Baron-Cohen et al. reported that AQ scores for students in the sciences (including mathematics) were significantly higher than those in the humanities and social sciences and that autistic traits were associated with scientific prowess [52]. The frequency of ALTs in the current study was close to that of computer science undergraduates, who had the highest frequency of ALTs in a study by Baron-Cohen et al. Autism has recently been reported to show significant positive shared heritability with one or more measures of cognitive function and academic achievement [65], and Japanese medical schools, where high school students with particularly high academic scores go, may experience more ALTs from students compared to other faculties. Although autistic traits were said to be more common in men than women [66], we did not observe significant gender differences in ALTs. Our results are in support of recent findings demonstrating that men are overrepresented in gender ratios and autistic traits are underdiagnosed in women [67–69].

### Limitations

This study has several limitations. First, this is a cross-sectional study and the causal relationship between ALTs and burnout or depression cannot be determined. However, ALTs are developmental traits, so logically, they could be a predictor of burnout and depression. Second, this study used self-administered questionnaires, which might have led to information bias. These questionnaires are valid, but they do not provide a definitive diagnosis. Third, the questionnaires were not answered by all medical students during clinical clerkships, which could lead to selection bias. We also lacked information on participant neurodiversity and mental health status, so we cannot rule out recruitment bias (i.e., participants with autistic traits or depression were more likely to enroll). However, this bias appears to be very small, as almost 80% of the medical students responded to the questionnaires. Fourth, we found significant gender differences in multivariate multiple linear regression with MBI-DP, MBI-PA, HADS-D, and HADS-Total as outcome variables. While this was probably due to effects from the participant gender ratio, we cannot rule out a true gender difference in the population. Fifh, this was a single-site study. Similar studies should be performed at other institutions in the future to accurately generalize these findings, although the hospital where this study was done shares similarities with other physician training facilities in Japan. Sixth, the causal relationship for the significant reduction in the prevalence of burnout and depression among medical students after COVID-19 is also not known because of the cross-sectional study. A longitudinal study is needed to examine temporal variance in prevalence of burnout and depression.

## Conclusion

This study examined the associations between ALTs and burnout or depression among medical students undergoing clinical clerkships in Japan. ALTs were significantly associated with lower personal accomplishment as a burnout factor and depression. These associations were mediated by certain factors in the psychological flexibility processes. Increased psychological flexibility is important for treating burnout and depression, but such a method requires consideration of the kinds of psychological flexibility processes to target, especially for medical students with ALTs.

## Supplementary Information

**Additional file 1: Table S1.** Demographic characteristics and descriptive statistical data categorized by gender.

**Additional file 2: Figure S1. **Illustration of the mediation model. Regression coefficients for each path are shown. Boot strap 95% confidence intervals are indicated in square brackets. Path γ1 indicates the effect of AQ-J-21 on MBI-PA without an indirect path. Path α1 shows the effect of AQ on VQ-P. Path β1 shows the effect of VQ-P on MBI-PA, with the effect of AQ-J-21 partially excluded. Path γ'1 (= γ1 - α1 * β1) is the direct effect of AQ-J-21 on MBI-PA. Path γ1 indicates the effect of AQ on HADS-D without an indirect path. Path α1 depicts the effect of AQ on CFQ-7. Path β1 shows the effect of CFQ-7 on HADS-D, with the effect of AQ partially excluded. Path δ1 denotes the effect of AQ on VQ-P. Path ε1 indicates the effect of VQ-P on HADS-D, and the effect of AQ is partially excluded. Pathway γ'1 (= γ1 - α1 * β1 - δ1 * ε1) is the direct effect of AQ on HADS-D. *p < 0.05, **p < 0.01.

## Data Availability

The datasets used and/or analyzed during the current study are available from the corresponding author upon reasonable request.

## Abbreviations

ACT: Acceptance and commitment therapy
AQ-J: Autism-Spectrum Quotient, Japanese version
CFQ-7: Cognitive Fusion Questionnaire-7
CI: Confidence interval
ICD-11: International Classification of Diseases
IQR: Interquartile Range
IRB: Institutional Review Board
MBI: Maslach Burnout Inventory
MBI-EE: Emotional Exhaustion Factor of Maslach Burnout Inventory
MBI-DP: Depersonalization Factor of Maslach Burnout Inventory
MBI-PA: Personal Accomplishment Factor of Maslach Burnout Inventory
OR: Odds ratio
SD: Standard deviation
VQ: Valuing Questionnaire
VQ-O: Obstruction Factor of Valuing Questionnaire
VQ-P: Progress Factor of Valuing Questionnaire
