# Supplementary material for: The mediating role of psychological flexibility in the association of autistic-like traits with burnout and depression in medical students during clinical clerkships in Japan: a university-based cross-sectional study
Source: BMC Psychiatry. 2023 May 1;23:302. doi: 10.1186/s12888-023-04811-y (PMC10150344; doi:10.1186/s12888-023-04811-y)
Supplement: Supplementary file 1 — Additional file 1: Table S1. Demographic characteristics and descriptive statistical data categorized by gender. [file 12888_2023_4811_MOESM1_ESM.docx]

Table S-1. Demographic characteristics and descriptive statistical data categorized by gender

| Variable | Total number (n = 226) | Gender (men) (n = 147) | Gender (women)  (n = 79) | *p*-value^*^ |
| --- | --- | --- | --- | --- |
|  |  | Number (%) |  |  |
| Before COVID | 61 (27.0) | 38 (25.8) | 23 (29.1) | 0.639 |
| After COVID | 165 (73.0) | 109 (74.1) | 56 ( 70.9) |  |
| High emotional exhaution^**^ | 65 (28.8) | 45 (30.6) | 20 ( 25.3) | 0.443 |
| High depersonalition ^**^ | **33 (14.6)** | **27 (18.4)** | **6 (7.6)** | **0.03** |
| Low personal accomplishment ^**^ | 198 (87.6) | 127 (86.4) | 71 ( 89.9) | 0.529 |
| Overall burnout ^**^ | 77 (34.1) | 55 (37.4) | 22 ( 27.8) | 0.185 |
| Highly anxious^***^ | 115 (50.9) | 73 (49.7) | 42 ( 53.2) | 0.676 |
| Highly depressed^***^ | 40 (17.7) | 30 (20.4) | 10 ( 12.7) | 0.2 |
| Highly anxious-depressed^***^ | 53 (23.5) | 40 (27.2) | 13 ( 16.5) | 0.073 |
| ALT ^a^ | 61 (27.0) | 41 (27.9) | 20 ( 25.3) | 0.754 |
| Alcohol consumption more than 3 times/week | 29 (12.8) | 17 (11.6) | 12 ( 15.2) | 0.532 |
| Participation in an extracurricular activity | 156 (69.0) | 98 (66.7) | 58 ( 73.4) | 0.366 |
| Presence of housemates | 90 (39.8) | 56 (38.1) | 34 ( 43.0) | 0.48 |
| Presence of a part-time job | 153 (67.7) | 94 (63.9) | 59 ( 74.7) | 0.104 |
| History of enrollment in other faculties | 15 (6.6) | 13 ( 8.8) | 2 (2.5) | 0.093 |
| Spouse | 4 (1.8) | 4 ( 2.7) | 0 (0.0) | 0.3 |
| Parenting | 4 (1.8) | 4 ( 2.7) | 0 (0.0) | 0.3 |
|  |  |  |  |  |
|  |  | Median [IQR^****^] |  |  |
| Age | 24.00  [23.00, 24.00] | 24.00　 [23.00, 24.00] | 23.00 [23.00, 24.00] | 0.121 |
| MBI-EE ^b^ | 17.00  [9.00, 29.00] | 19.00  [9.00, 29.50] | 16.00  [9.50, 27.00] | 0.46 |
| MBI-DP ^c^ | 2.00  [0.00, 6.00] | 2.00  [0.00, 8.00] | 1.00  [0.00, 4.00] | 0.084 |
| MBI-PA ^d^ | 22.00  [15.00, 29.00] | **23.00  [17.00, 30.00]** | **20.00  [12.00, 26.00]** | **0.008** |
| HADS-A ^g^ | **8.00**  **[5.00, 10.00]** | 7.00  [4.50, 10.50] | 8.00  [5.00, 9.00] | 0.883 |
| HADS-D ^f^ | **6.00**  **[4.00, 9.75]** | **7.00  [4.50, 10.00]** | **5.00 [3.50, 8.00]** | **0.003** |
| HADS-Total ^e^ | 14.00  [9.00, 19.00] | 15.00  [9.00, 20.00] | 13.00  [9.50, 17.00] | 0.108 |
| AQ-J-21 ^h^ | 9.00  [6.00, 12.00] | 9.00  [6.00, 12.00] | 9.00  [6.50, 11.50] | 0.854 |
| CFQ-7 ^i^ | 24.00  [17.00, 29.00] | 24.00  [17.00, 30.50] | 24.00  [18.50, 29.00] | 0.807 |
| VQ -P ^j^ | 19.00  [15.00, 23.00] | 20.00  [15.00, 24.00] | 19.00  [16.00, 21.50] | 0.293 |
| VQ-O ^k^ | 17.00  [14.00, 21.00] | 17.00  [14.00, 21.00] | 18.00  [14.50, 20.50] | 0.597 |

^*^ Chi-square tests for categorical data. Mann-Whitney U test for numerical data. Bold text indicates statistical significance between before and after the COVID-19 pandemic at *p* < 0.05.

^**^ MBI-EE score of ≥27 was defined as high emotional exhaustion, MBI-DP score of ≥ 10 as high depersonalization, MBI-PA score of ≤33 as low degree of personal accomplishment, and MBI-EE score of ≥27 and/or MBI-EE score of ≥10 as overall burnout.

^***^ A score of ≥ 8 on the HADS Anxiety subscale was categorized as highly anxious, a score of ≥ 11 on the HADS Depression subscale as highly depressed, and a total score of ≥ 20 as highly anxious-depressed.

^****^ IQR: Interquartile Range.

^a^ ALT: Autistic-like trait; ^b^ MBI-EE: Maslach Burnout Inventory-Emotional Exhaustion; ^c^ MBI-DP: Maslach Burnout Inventory-Depersonalization; ^d^ MBI-PA: Maslach Burnout Inventory-Personal Accomplishment; ^e^ HADS-A: Hospital Anxiety and Depression Scale-Anxiety subscale score; ^f^ HADS-D: Hospital Anxiety and Depression Scale-Depression subscale score; ^g^ HADS-Total: Hospital Anxiety and Depression Scale-Total score; ^h^ AQ-J-21: Autism-Spectrum Quotient, Japanese version -21 ; ^i^ CFQ-7: Cognitive Fusion Questionnaire-7; ^j^ VQ-P: Valuing Questionnaire-Progress; ^k^ VQ-O: Valuing Questionnaire-Obstruction
