# Supplementary material for: The mediating role of psychological flexibility in the association of autistic-like traits with burnout and depression in medical students during clinical clerkships in Japan: a university-based cross-sectional study
Source: BMC Psychiatry. 2023 May 1;23:302. doi: 10.1186/s12888-023-04811-y (PMC10150344; doi:10.1186/s12888-023-04811-y)
Supplement: Supplementary file 2 — Additional file 2: Figure S1. Illustration of the mediation model. Regression coefficients for each path are shown. Boot strap 95% confidence intervals are indicated in square brackets. Path γ1 indicates the effect of AQ-J-21 on MBI-PA without an indirect path. Path α1 shows the effect of AQ on VQ-P. Path β1 shows the effect of VQ-P on MBI-PA, with the effect of AQ-J-21 partially excluded. Path γ'1 (= γ1 - α1 * β1) is the direct effect of AQ-J-21 on MBI-PA. Path γ1 indicates the effect of AQ on HADS-D without an indirect path. Path α1 depicts the effect of AQ on CFQ-7. Path β1 shows the effect of CFQ-7 on HADS-D, with the effect of AQ partially excluded. Path δ1 denotes the effect of AQ on VQ-P. Path ε1 indicates the effect of VQ-P on HADS-D, and the effect of AQ is partially excluded. Pathway γ'1 (= γ1 - α1 * β1 - δ1 * ε1) is the direct effect of AQ on HADS-D. *p < 0.05, **p < 0.01. [file 12888_2023_4811_MOESM2_ESM.docx]

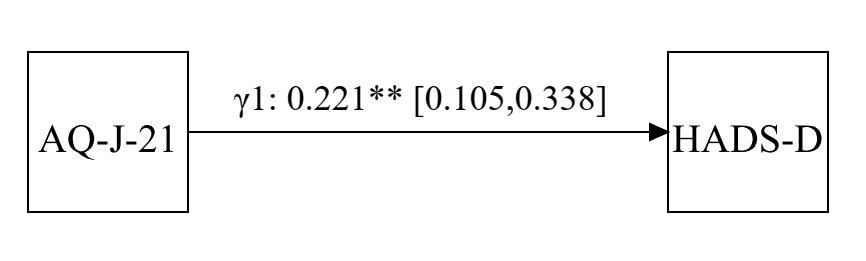


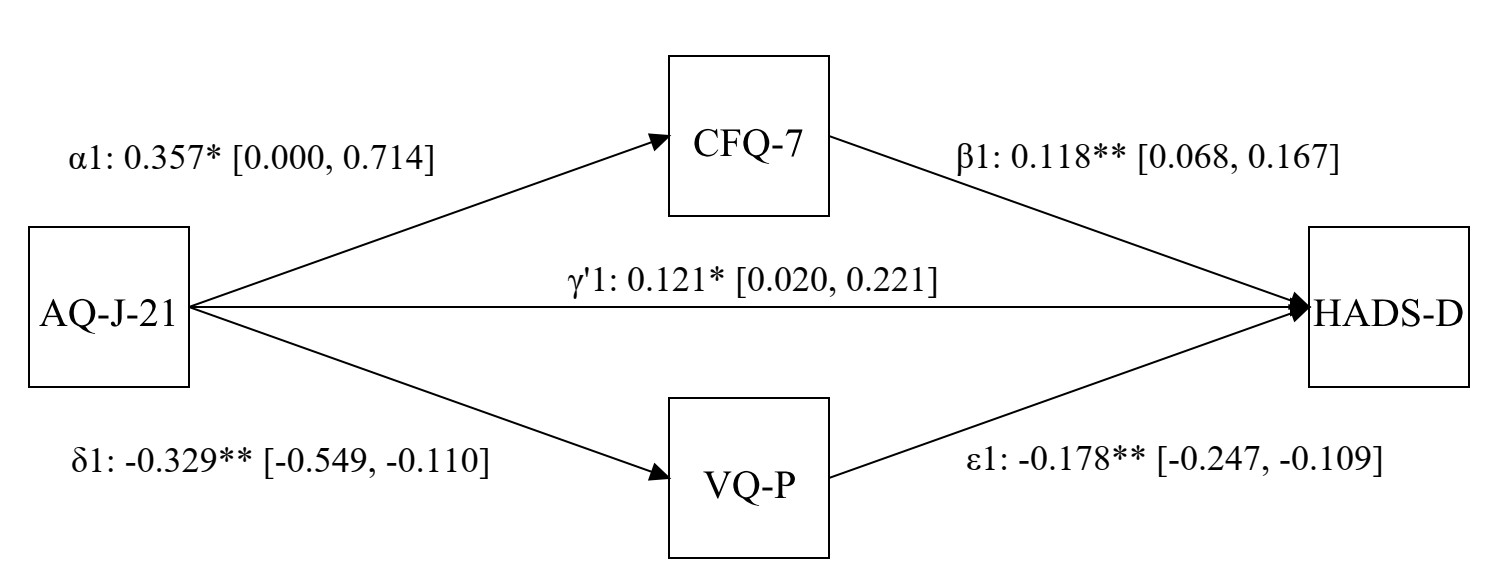


**Figure S1** Illustration of the mediation model. Regression coefficients for each path are shown. Boot strap 95% confidence intervals are indicated in square brackets. Path γ1 indicates the effect of AQ-J-21 on MBI-PA without an indirect path. Path α1 shows the effect of AQ on VQ-P. Path β1 shows the effect of VQ-P on MBI-PA, with the effect of AQ-J-21 partially excluded. Path γ'1 (= γ1 - α1 * β1) is the direct effect of AQ-J-21 on MBI-PA. Path γ1 indicates the effect of AQ on HADS-D without an indirect path. Path α1 depicts the effect of AQ on CFQ-7. Path β1 shows the effect of CFQ-7 on HADS-D, with the effect of AQ partially excluded. Path δ1 denotes the effect of AQ on VQ-P. Path ε1 indicates the effect of VQ-P on HADS-D, and the effect of AQ is partially excluded. Pathway γ'1 (= γ1 - α1 * β1 - δ1 * ε1) is the direct effect of AQ on HADS-D. **p* < 0.05, ***p* < 0.01.
